# Author Correction: Multiparameter prediction of myeloid neoplasia risk

**DOI:** 10.1038/s41588-023-01532-6

**Published:** 2023-09-19

**Authors:** Muxin Gu, Sruthi Cheloor Kovilakam, William G. Dunn, Ludovica Marando, Clea Barcena, Irina Mohorianu, Alexandra Smith, Siddhartha P. Kar, Margarete A. Fabre, Moritz Gerstung, Catherine A. Cargo, Luca Malcovati, Pedro M. Quiros, George S. Vassiliou

**Affiliations:** 1 Wellcome-MRC Cambridge Stem Cell Institute, University of Cambridge, Cambridge, UK; 2 Department of Haematology, University of Cambridge, Cambridge, UK; 3 Department of Biochemistry and Molecular Biology, Universidad de Oviedo, Oviedo, Spain; 4 Epidemiology and Cancer Statistics Group, University of York, York, UK; 5 MRC Integrative Epidemiology Unit, University of Bristol, Bristol, UK; 6 Section of Translational Epidemiology, Division of Population Health Sciences, Bristol, Medical School, University of Bristol, Bristol, UK; 7 Early Cancer Institute, Department of Oncology, University of Cambridge, Cambridge, UK; 8 Centre for Genomics Research, Discovery Sciences, BioPharmaceuticals R&D, AstraZeneca, Cambridge, UK; 9 Division of Artificial Intelligence in Oncology, DKFZ, Heidelberg, Germany; 10 Haematological Malignancy Diagnostic Service, St James’s Hospital, Leeds, UK; 11 Department of Haematology, Leeds Teaching Hospitals, Leeds, UK; 12 Department of Molecular Medicine, University of Pavia, Pavia, Italy; 13 Department of Hematology, Fondazione IRCCS Policlinico San Matteo, Pavia, Italy; 14 Instituto de Investigación Sanitaria del Principado de Asturias, ISPA, Oviedo, Spain; 15 Department of Haematology, Cambridge University Hospitals NHS Trust, Cambridge, UK

**Keywords:** Haematological cancer, Genetics research, Outcomes research

Correction to: *Nature Genetics* 10.1038/s41588-023-01472-1, published online 24 August 2023.

In the version of the article initially published, grants to L.M. from the Associazione Italiana per la Ricerca sul Cancro (AIRC) (grant 20125; AIRC 5x1000 project 21267) and Cancer Research UK, FC AECC and AIRC under the International Accelerator Award Program (C355/A26819 and 22796) were missing from the Acknowledgments section. The grant information has been inserted in the HTML and PDF versions of the article.

