# Supplementary material for: Multiparameter prediction of myeloid neoplasia risk
Source: Nat Genet. 2023 Aug 24;55(9):1523–30. doi: 10.1038/s41588-023-01472-1 (PMC10484784; doi:10.1038/s41588-023-01472-1)
Supplement: Supplementary file 1 — Supplementary Figs. 1–11. [file 41588_2023_1472_MOESM1_ESM.pdf]

nature genetics

Article https://doi.org/10.1038/s41588-023-01472-1

Multiparameter prediction of myeloid neoplasia risk

In the format provided by the
authors and unedited

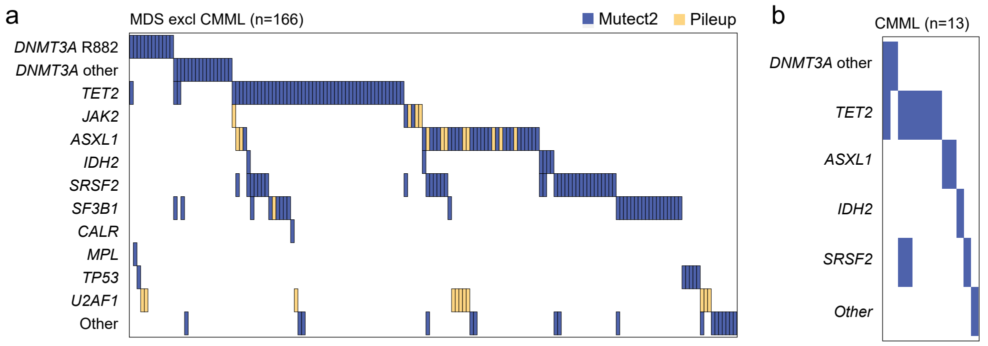

Supplementary Fig. 1: Pre-MDS and Pre-CMML in the UKB display similar CH mutation profiles. Waterfall plot outlining the mutation profiles of **(a)** 166 pre-MDS **(b)** and 13 pre-CMML cases with at least one mutation. Mutations identified by Mutect2 are depicted in blue and those identified by Samtools mpileup in yellow.

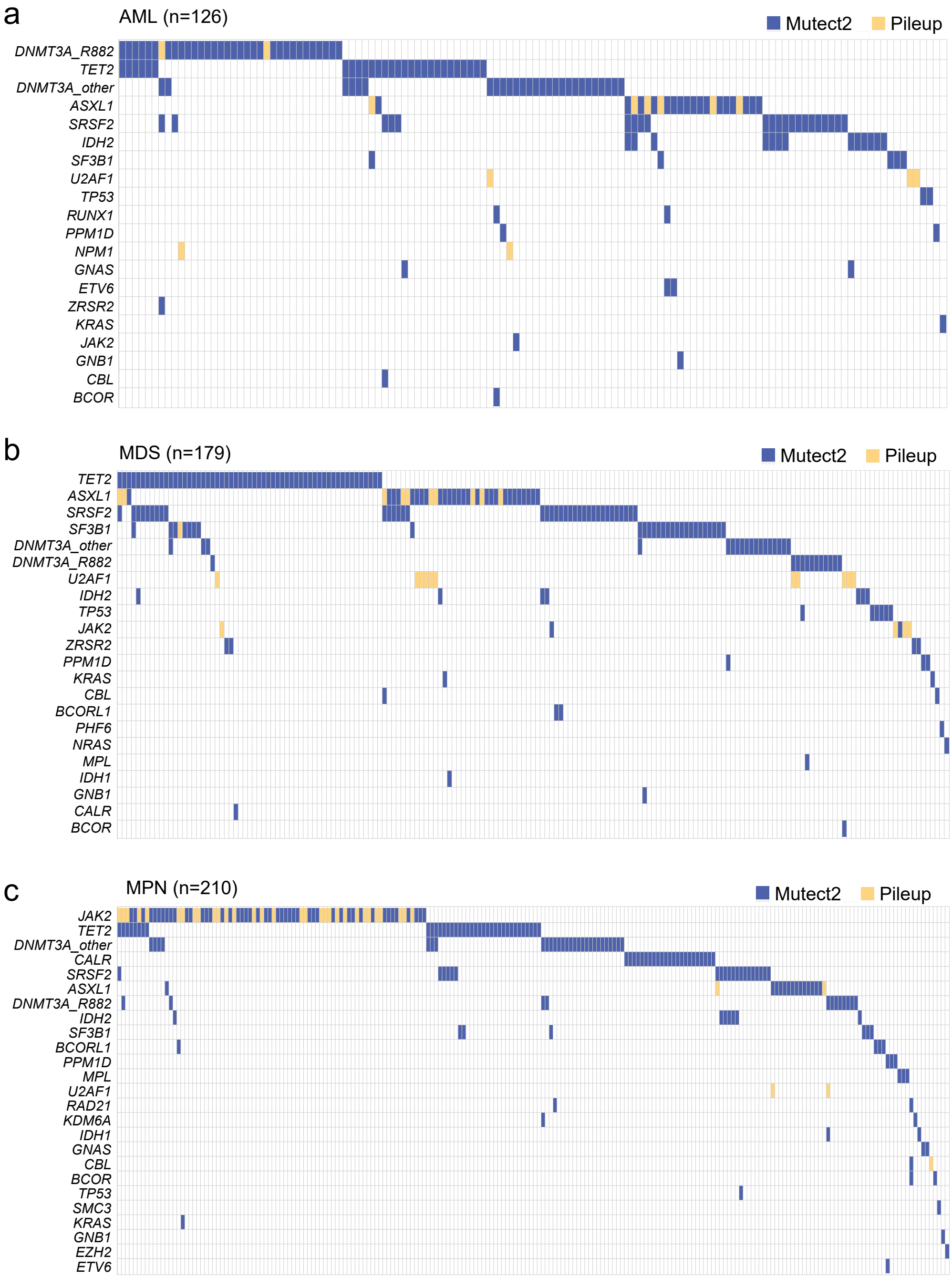

Supplementary Fig. 2: Full CH mutation profiles in pre-AML, pre-MDS and pre-MPN in the UKB. Waterfall plots depicting all nutation in 38 CH genes (Supplementary Table 1) amongst all individuals with pre-MN in the UKB, namely **(a)** 126 cases of pre-AML, **(b)** 179 cases of pre-MDS, including pre-CMML, and **(c)** 210 cases of pre-MPN. Mutations identified by Mutect2 are depicted in blue and those identified by Samtools mpileup in yellow.

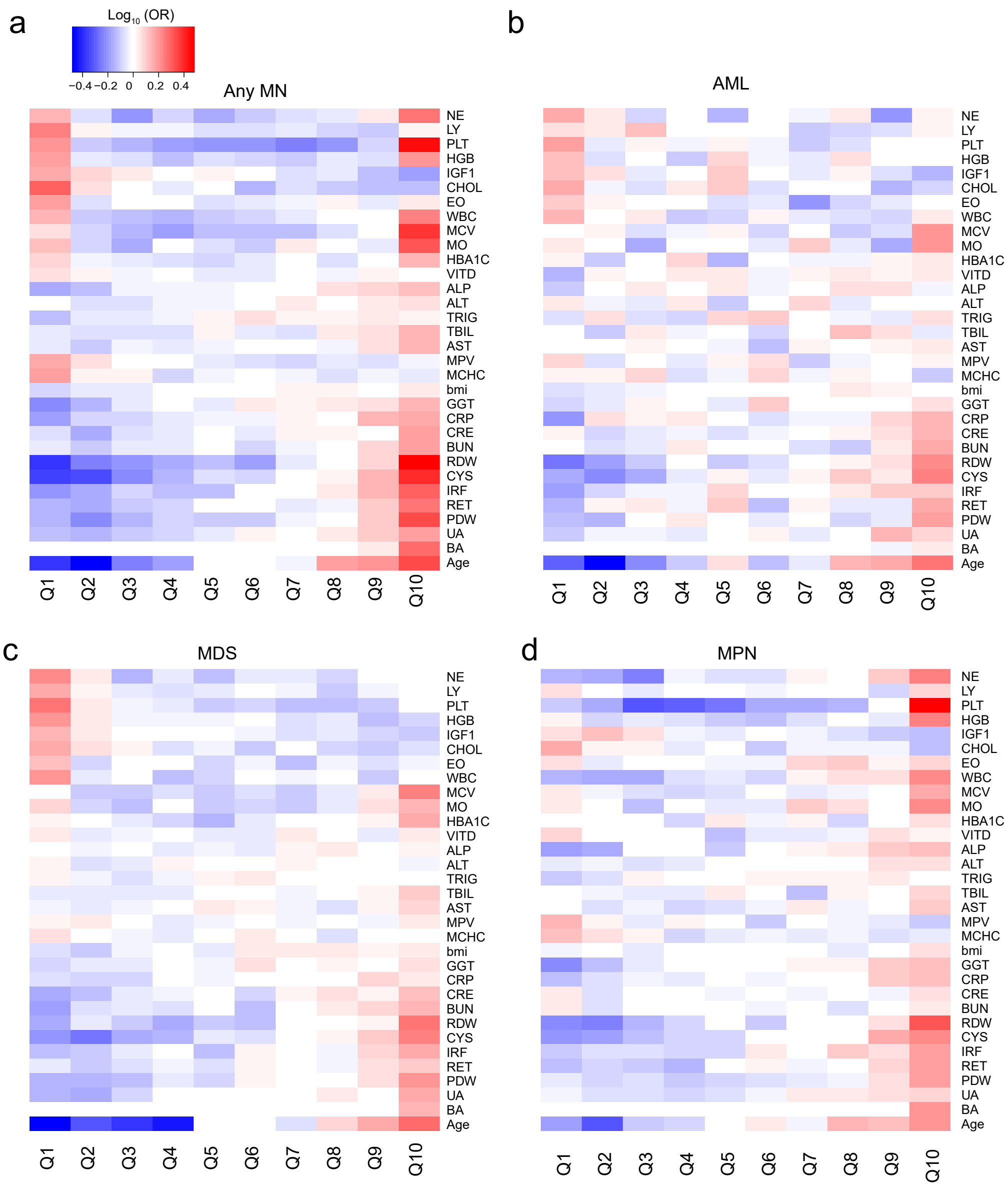

Supplementary Fig. 3: Trends in blood test parameters in different types of pre-MN. Depiction of trends in the values of different hematology (CBC) and biochemistry parameters in all cases of pre-MN **(a)** and in each of the three pre-MN subtypes individually, namely **(b)** pre-AML **(c)** pre-MDS and **(d)** pre-MPN. Values for each parameter from the entire UKB cohort were divided into deciles (Q1 to Q10) and the overlap between the relevant pre-MN type and each quantile is calculated and displayed as the log10 of odds ratio (OR), with shades of blue depicting depletion and shades of red depicting enrichment. For example, there are more AMLs than expected by chance in Q8-10 of RDW values and fewer in Q1-4 (b). This reveals significant differences between pre-MN subtypes; for example looking at platelet counts (PLT), pre-AML (b) and pre-MDS (c) are more likely to have very low (Q1), whilst pre-MPN (d) are more likely to have very high counts (Q10).

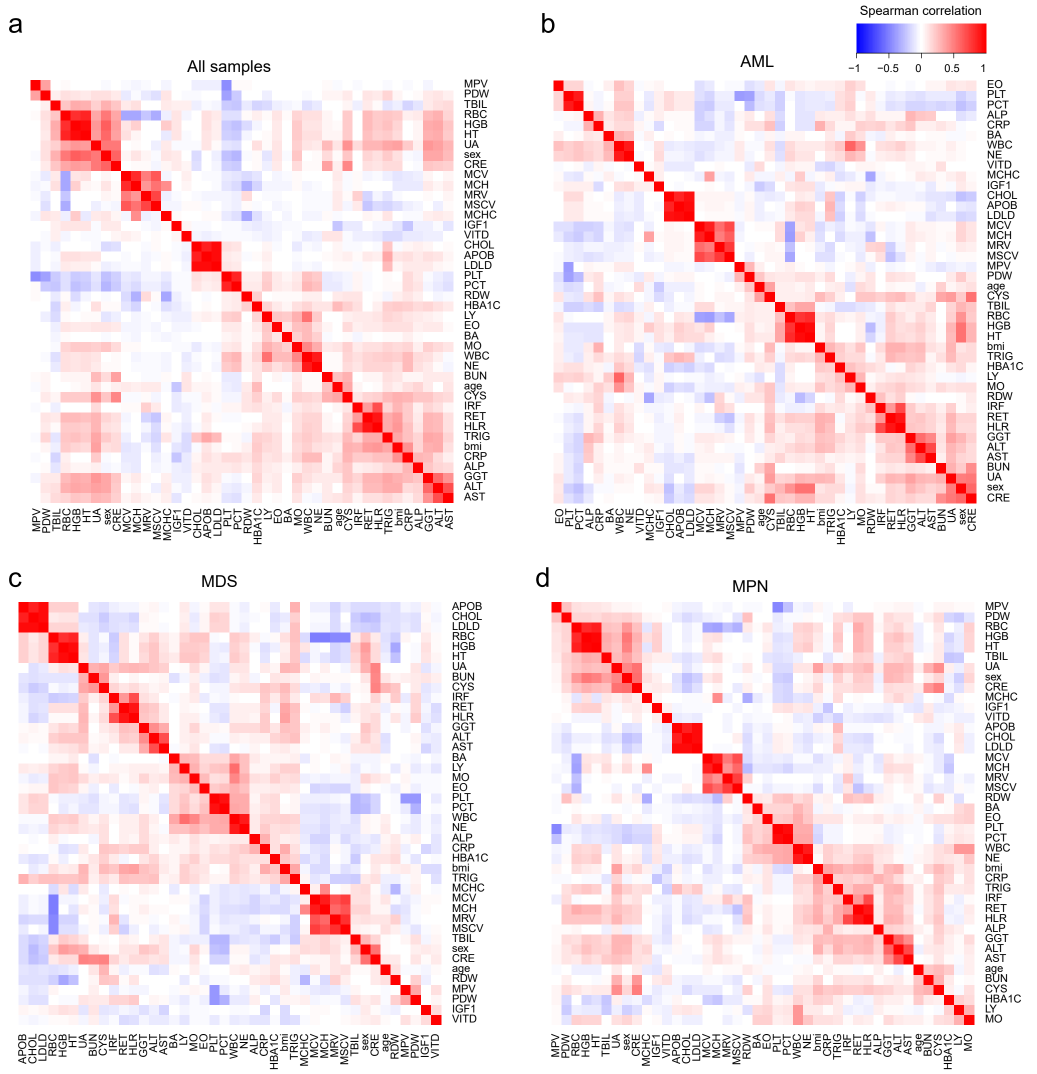

Supplementary Fig. 4: Correlations between results for blood test parameters in the UKB. Correlation plots depicting Spearman correlation coefficients between different blood cell count and biochemistry results in: **(a)** entire UKB, **(b)** 372 pre-AML, **(c)** 544 pre-MDS and (D) 892 pre-MPN cases. Highly correlated parameters (Spearman Correlation>0.9) were collapsed to a single parameter (also see Methods).

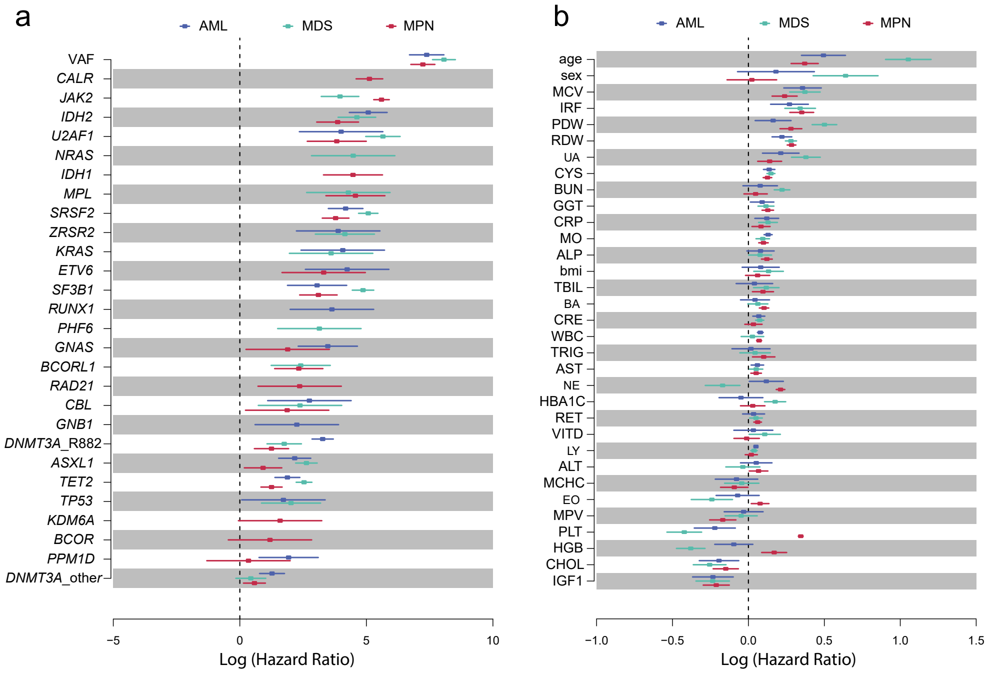

Supplementary Fig. 5: Univariate Cox hazard ratios for developing MN associated with different parameters. **(a)** Hazard ratios (HR) for different types of MN associated with mutations in any of the 38 genes (only genes mutated in $\geq$2 cases are depicted). *DNMT3A* R882 mutations are depicted separately from other mutations in this gene. The impact of clonal size (variant allele fraction, VAF) is also shown independently of the mutated gene. **(b)** HRs associated with age, sex and 30 blood test parameters HRs were calculated using univariate Cox proportional hazard models for AML, MDS and MPN risk. See Supplementary Table 4 for abbreviations. The central squares indicate hazard ratios and the lines indicate 5-95% confident intervals.

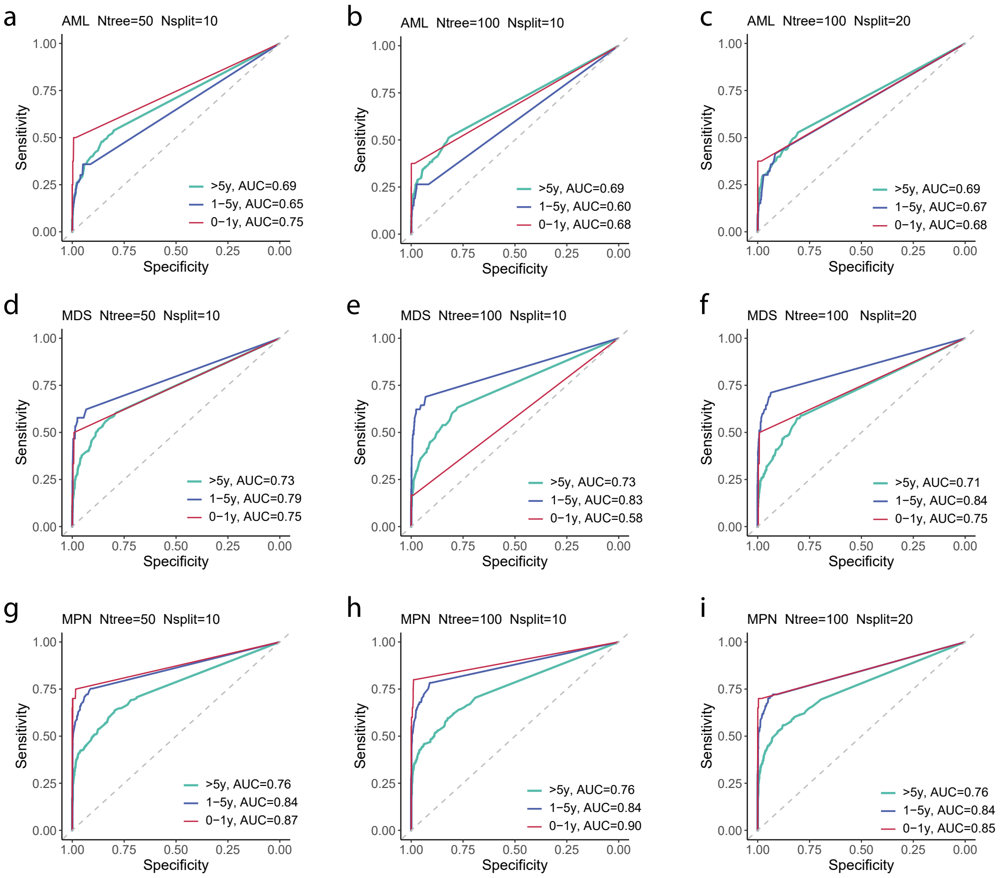

Supplementary Fig. 6: Assessment of prediction accuracy of random survival forest models using various parameters. Time-dependent Receiver Operating Characteristics (ROC) curves computed from predicted outcomes on the validation set at various time intervals versus clinical diagnosis of individuals who developed MN in 0-1 year, 1-5 years and over 5 years after samples were taken. ROC curves were computed using the incident/dynamic method (see Methods for details, AUC=area under curve, Ntree=number of trees, Nsplit=maximum tree split). **(a-c)** AML prediction, **(d-f)** MDS prediction, **(g-i)** MPN prediction.

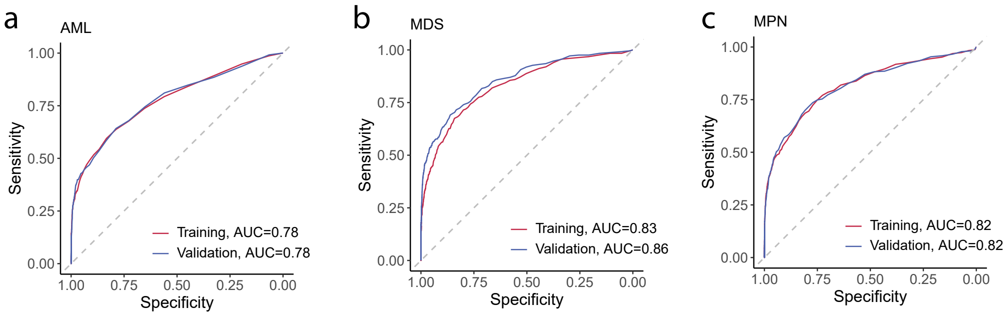

Supplementary Fig. 7: Performance of Cox-based models on training and validation sets. Receiver Operating Characteristics (ROC) curves computed from predicted probability on the training/validation set of developing MN in 15 years, and clinical diagnosis of MN within 15 years after blood sampling. AUC=area under curve. **(a)** Prediction of AML cases. **(b)** Prediction of MDS cases. **(c)** Prediction of MPN cases.

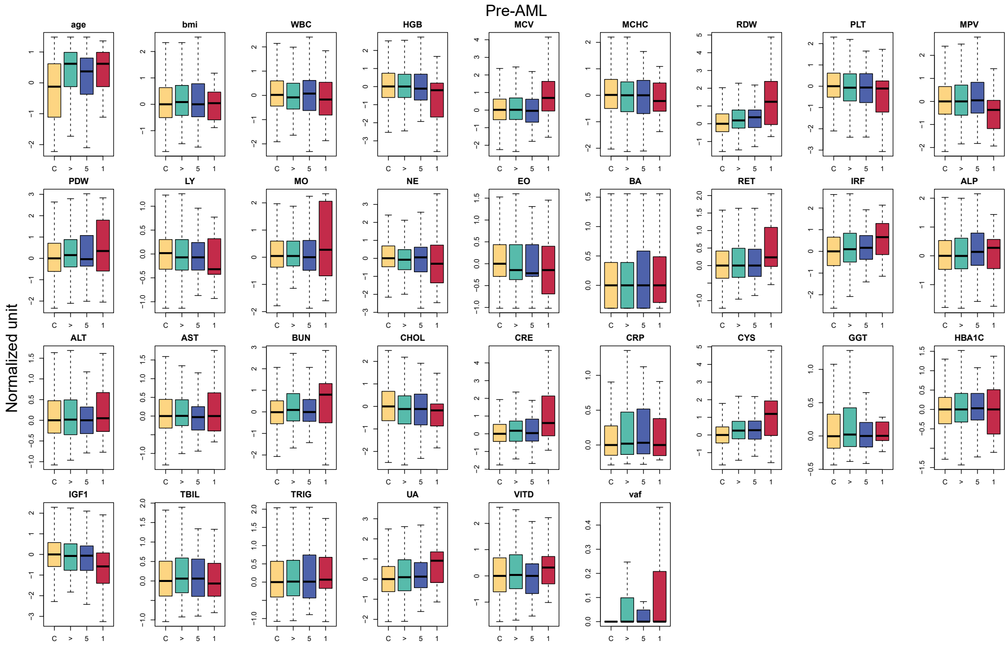

Supplementary Fig. 8: Full set of hematology and biochemistry results by time to AML diagnosis. Impact of time from diagnosis on the distribution of all blood and biochemistry parameters, age, BMI and mutation VAF in pre-AML. Box plots are color coded as follows: Controls ('C') in yellow, pre-AML samples taken >5 years before diagnosis ('>') in green, 1-5 years ('5') in blue and 0-1 ('1') years in red. In the box plots, central lines indicate medians, boxes indicate 25-75% quantiles and ranges indicate 1.5 interquartile ranges from the upper or lower quartiles.

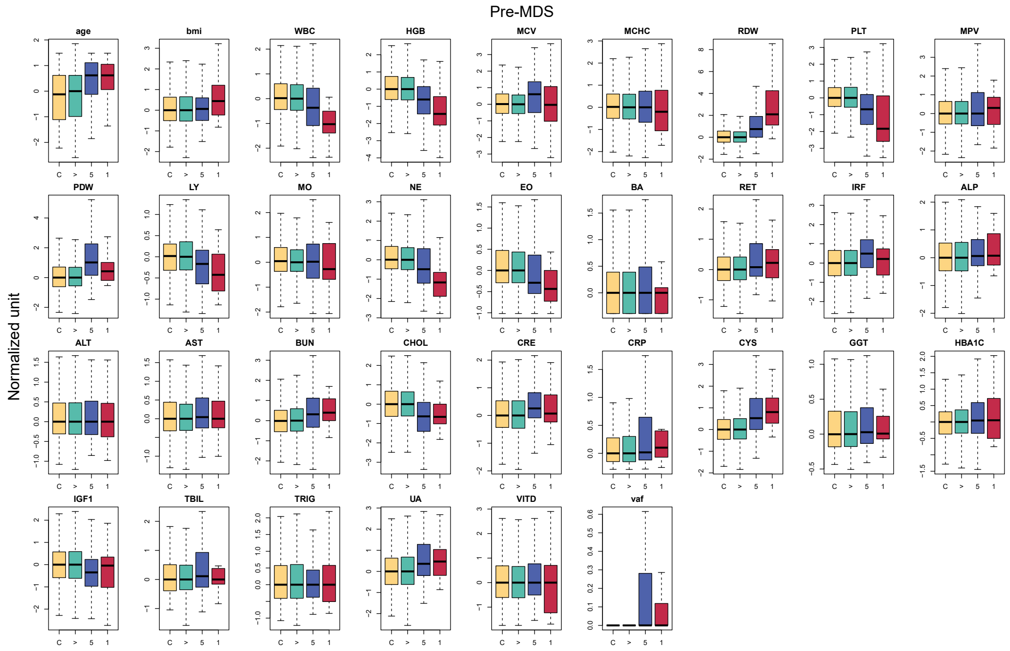

Supplementary Fig. 9: Full set of hematology and biochemistry results by time to MDS diagnosis. Impact of time from diagnosis on the distribution of all blood and biochemistry parameters, age, BMI and mutation VAF in pre-AML. Box plots are color coded as follows: Controls ('C') in yellow, pre-MDS samples taken >5 years before diagnosis ('>') in green, 1-5 years ('5') in blue and 0-1 ('1') years in red. In the box plots, central lines indicate medians, boxes indicate 25-75% quantiles and ranges indicate 1.5 interquartile ranges from the upper or lower quartiles.

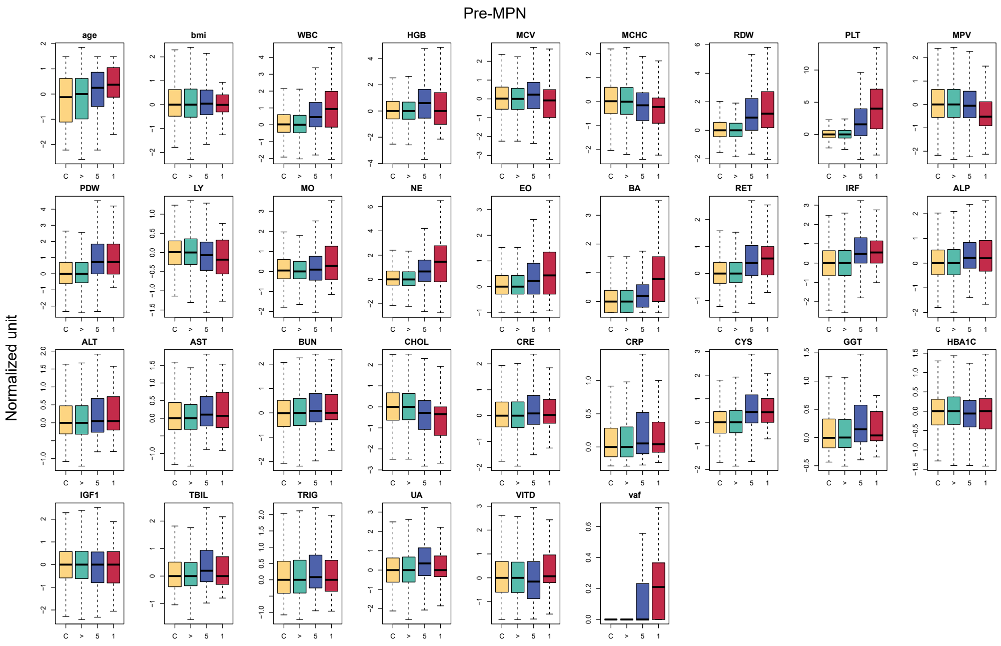

Supplementary Fig. 10: Full set of hematology and biochemistry results by time to MPN diagnosis. Impact of time from diagnosis on the distribution of all blood and biochemistry parameters, age, BMI and mutation VAF in pre-AML. Box plots are color coded as follows: Controls (’C’) in yellow, pre-MPN samples taken >5 years before diagnosis (’¿’) in green, 1-5 years (’5’) in blue and 0-1 (’1’) years in red. In the box plots, central lines indicate medians, boxes indicate 25-75% quantiles and ranges indicate 1.5 interquartile ranges from the upper or lower quartiles.

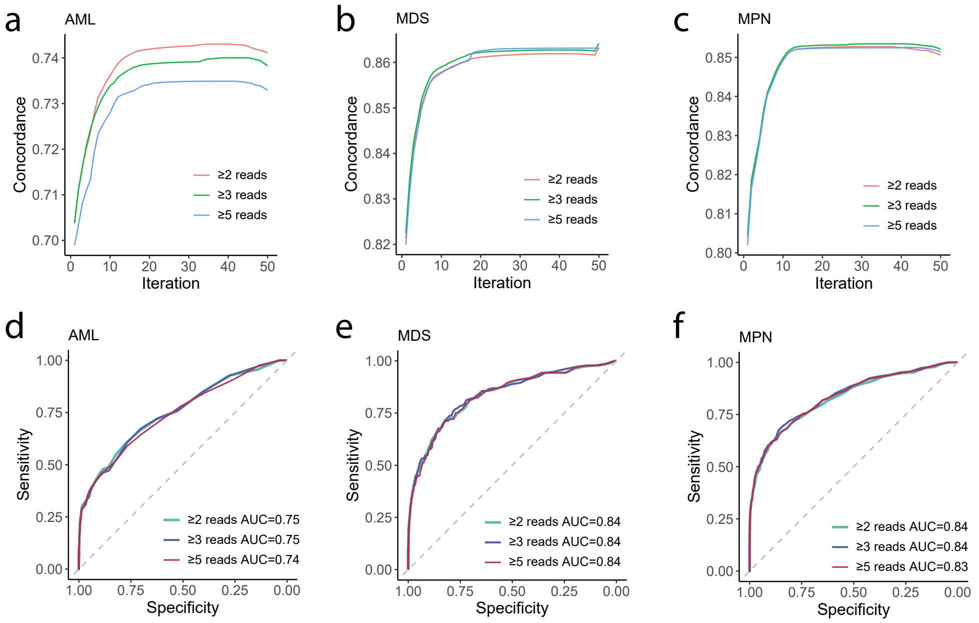

Supplementary Fig. 11: Effect of mutant read cut-offs on model training and performance. (a-c) Differences in concordance index of stepwise Cox regressions trained from Mutect2 calls that were filtered by ≥ 2, ≥ 3 or ≥ 5 mutant reads, for **(a)** AML, **(b)** MDS and **(c)** MPN. Details refer to Methods. **(d-f)** Comparison of ROC curves, computed on the validation set, of the Cox regression models trained from Mutect2 calls that were filtered by ≥ 2, ≥ 3 or ≥ 5 mutant reads, for **(d)** AML, **(e)** MDS and **(f)** MPN. AUC = area under curve.
