# Supplementary material for: Multiparameter prediction of myeloid neoplasia risk
Source: Nat Genet. 2023 Aug 24;55(9):1523–30. doi: 10.1038/s41588-023-01472-1 (PMC10484784; doi:10.1038/s41588-023-01472-1)
Supplement: Supplementary file 2 — Reporting Summary [file 41588_2023_1472_MOESM2_ESM.pdf]

nature portfolio

Corresponding author(s): eorge S. Vassiliou & Pedro Quiros

Last updated by author(s): May 4, 2023

Reporting Summary

Nature Portfolio wishes to improve the reproducibility of the work that we publish. This form provides structure for consistency and transparency in reporting. For further information on Nature Portfolio policies, see our Editorial Policies and the Editorial Policy Checklist.

Statistics

For all statistical analyses, confirm that the following items are present in the figure legend, table legend, main text, or Methods section.

n/a	Confirmed	
☐	☒	The exact sample size (*n*) for each experimental group/condition, given as a discrete number and unit of measurement
☒	☐	A statement on whether measurements were taken from distinct samples or whether the same sample was measured repeatedly
☐	☒	The statistical test(s) used AND whether they are one- or two-sided<br>*Only common tests should be described solely by name; describe more complex techniques in the Methods section.*
☐	☒	A description of all covariates tested
☒	☐	A description of any assumptions or corrections, such as tests of normality and adjustment for multiple comparisons
☐	☒	A full description of the statistical parameters including central tendency (e.g. means) or other basic estimates (e.g. regression coefficient) AND variation (e.g. standard deviation) or associated estimates of uncertainty (e.g. confidence intervals)
☐	☒	For null hypothesis testing, the test statistic (e.g. F, t, r) with confidence intervals, effect sizes, degrees of freedom and P value noted<br>*Give P values as exact values whenever suitable.*
☒	☐	For Bayesian analysis, information on the choice of priors and Markov chain Monte Carlo settings
☒	☐	For hierarchical and complex designs, identification of the appropriate level for tests and full reporting of outcomes
☐	☒	Estimates of effect sizes (e.g. Cohen's *d*, Pearson's *r*), indicating how they were calculated

Our web collection on statistics for biologists contains articles on many of the points above.

Software and code

Policy information about availability of computer code

Data collection	No raw data was downloaded. Analysis was performed using UKB data accessed via DNA Nexus (ukbiobank.dnanexus.com)
Data analysis	GATK v4.1.3.0, Mutect2. Samtools 1.15. Custom code for training Cox regression models can be found here: github.com/muxingu/mnpredict_paper.

For manuscripts utilizing custom algorithms or software that are central to the research but not yet described in published literature, software must be made available to editors and reviewers. We strongly encourage code deposition in a community repository (e.g. GitHub). See the Nature Portfolio guidelines for submitting code & software for further information.

Data

Policy information about availability of data

All manuscripts must include a data availability statement. This statement should provide the following information, where applicable:

- Accession codes, unique identifiers, or web links for publicly available datasets
- A description of any restrictions on data availability
- For clinical datasets or third party data, please ensure that the statement adheres to our policy

Provide your data availability statement here.

Research involving human participants, their data, or biological material

Policy information about studies with human participants or human data. See also policy information about sex, gender (identity/presentation), and sexual orientation and race, ethnicity and racism.

Reporting on sex and gender	We used sex as a variable in our regression/predictive models as male sex is associated with an increased risk of myeloid neoplasia. Individual level information on sex is available from the UKB.
Reporting on race, ethnicity, or other socially relevant groupings	The UK Biobank is primarily populated by individuals of European ancestry (~85%).
Population characteristics	The UK Biobank is a prospective longitudinal study containing in-depth genetic and health information from ~half a million UK participants. For this study, we analysed 454,340 individuals who had whole-exome sequencing (WES) data released as of March 2022 (age range: 38-72, mean age: 56.5; 54.1% female; ~83% White British).
Recruitment	As stated above, the UK Biobank is a prospective longitudinal study containing in-depth genetic and health information from ~500,000 UK participants. Details of UK Biobank participant recruitment are available at: https://www.ukbiobank.ac.uk and from Sudlow C, et al. (2015) PLoS Med 12(3): e1001779. For this study, we analyzed 454,340 individuals who had whole-exome sequencing (WES) data released as of March 2022. Notably, participants were not selected in any way, however as is the case for several such cohorts, there is evidence of selection bias in favor of healthier, older, female, socio-economically better off volunteers (Fry A, et al. Am. J. of Epidemiol., Vol. 186, Issue 9, 1 Nov. 2017, Pgs. 1026–1034). Also, despite a relatively low response rate to invitations to participate, it has been shown that risk factor associations identified in the UK Biobank are generalizable (Batty GD, et al. BMJ 2020; 368:m131).
Ethics oversight	The UK Biobank study has been approved by the North West Multicentre Research Ethics Committee (11/NW/0382). All participants provided written informed consent to the UK Biobank. Further details can be found at: https://www.ukbiobank.ac.uk/learn-more-about-uk-biobank/about-us/ethics. The current study has been conducted under approved UK Biobank application numbers 56844.

Note that full information on the approval of the study protocol must also be provided in the manuscript.

Field-specific reporting

Please select the one below that is the best fit for your research. If you are not sure, read the appropriate sections before making your selection.

☒ Life sciences ☐ Behavioural & social sciences ☐ Ecological, evolutionary & environmental sciences

For a reference copy of the document with all sections, see nature.com/documents/nr-reporting-summary-flat.pdf

Life sciences study design

All studies must disclose on these points even when the disclosure is negative.

Sample size	For this study, we analyzed 454,340 individuals who had whole-exome sequencing (WES) data released as of March 2022.
Data exclusions	Participants that developed myeloid neoplasia prior to recruitment were excluded from model training & validation. Also, individuals with more than two missing values in their blood cell count or biochemistry datasets were excluded. We also removed 108 UKB participants with blood test results consistent with a diagnosis of MPN at the time of recruitment from model training and validation.
Replication	We divided the 414,074 eligible (non-excluded) participants into a training set of 207,035 and a validation set of 207,039. Models were trained on the training set and validated on the validation set.
Randomization	The division of participants into training and validation sets was random and performed using the Math.random() function from Java.
Blinding	Not applicable, as the analyses presented in this manuscript do not include any intervention or clinical trial + learning used in our models was supervised (i.e status re myeloid neoplasia had to be known).

Reporting for specific materials, systems and methods

We require information from authors about some types of materials, experimental systems and methods used in many studies. Here, indicate whether each material, system or method listed is relevant to your study. If you are not sure if a list item applies to your research, read the appropriate section before selecting a response.

Materials & experimental systems

n/a	Involved in the study
☒	☐ Antibodies
☒	☐ Eukaryotic cell lines
☒	☐ Palaeontology and archaeology
☒	☐ Animals and other organisms
☐	☒ Clinical data
☒	☐ Dual use research of concern
☒	☐ Plants

Methods

n/a	Involved in the study
☒	☐ ChIP-seq
☒	☐ Flow cytometry
☒	☐ MRI-based neuroimaging

Clinical data

Policy information about clinical studies

All manuscripts should comply with the ICMJE guidelines for publication of clinical research and a completed CONSORT checklist must be included with all submissions.

Clinical trial registration: N/A

Study protocol: N/A

Data collection: Clinical outcome data on myeloid neoplasia development was collected by the UK Biobank and by Drs Malcovati (Pavia CCUS cohort) and Drs Cargo & Smith (Leeds cohort)

Outcomes: Clinical outcome data on myeloid neoplasia development was collected by the UK Biobank and by Drs Malcovati (Pavia CCUS cohort) and Drs Cargo & Smith (Leeds cohort)
